# Isoprostanes and Isofurans in Infertility and Assisted Reproduction: What Do We Know So Far?

**DOI:** 10.3390/ijms27114710

**Published:** 2026-05-23

**Authors:** Charalampos Voros, Fotios Chatzinikolaou, Georgios Papadimas, Athanasios Karpouzos, Aristotelis-Marios Koulakmanidis, Diamantis Athanasiou, Kyriakos Bananis, Antonia Athanasiou, Aikaterini Athanasiou, Charalampos Tsimpoukelis, Ioannis Papapanagiotou, Maria Anastasia Daskalaki, Christina Trakateli, Nana Kojo Koranteng, Nikolaos Thomakos, Panagiotis Antsaklis, Dimitrios Loutradis, Georgios Daskalakis

**Affiliations:** 1 Department of Obstetrics and Gynecology, ‘Alexandra’ General Hospital, National and Kapodistrian University of Athens, 80 Vasilissis Sofias Avenue, 11528 Athens, Greece; thanoskarpouzosdr@hotmail.com (A.K.); aristoteliskoulak@gmail.com (A.-M.K.); tsimpoukelischa@gmail.com (C.T.); md181341@students.euc.ac.cy (M.A.D.); thomakir@hotmail.com (N.T.); panosant@gmail.com (P.A.); 2 Laboratory of Forensic Medicine and Toxicology, School of Medicine, Aristotle University of Thessaloniki, 54124 Athens, Greece; fotischatzin@auth.gr; 3 Athens Medical School, National and Kapodistrian University of Athens, 15772 Athens, Greece; dr.georgepapadimas@gmail.com (G.P.); diamathan16@gmail.com (D.A.); antoathan16@gmail.com (A.A.); diamathan17@gmail.com (A.A.); gpapamd@hotmail.com (I.P.); kojo.k@icloud.com (N.K.K.); loutradi@otenet.gr (D.L.); 4 King’s College Hospitals NHS Foundation Trust, London SE5 9RS, UK; kyriakos.bananis@nhs.net; 5 3rd Department of Internal Medicine, Aristotle University Thessaloniki, 56403 Thessaloniki, Greece; ctrak@auth.gr

**Keywords:** oxidative stress, lipid peroxidation, reproductive biology, follicular fluid, seminal plasma, mitochondrial dysfunction, arachidonic acid metabolism, assisted reproductive technologies, sperm function, oocyte quality

## Abstract

Oxidative stress is a fundamental mechanism that impacts reproductive function by altering gamete quality, fertilisation, and the initial development of embryos. Excessive reactive oxygen species lead to the oxidation of polyunsaturated fatty acids in the cell membranes of sperm, oocytes, and adjacent somatic cells. F2-isoprostanes and isofurans are two of the most dependable indicators of oxidative lipid damage among the byproducts generated during free radical-mediated lipid oxidation. Both arise from the non-enzymatic peroxidation of arachidonic acid and provide a chemically stable depiction of in vivo oxidative processes. Reproductive studies indicate that elevated levels of F2-isoprostanes are associated with diminished sperm motility, compromised membrane stability, and an increased risk of DNA fragmentation in various forms of male infertility. Lipid peroxidation products have been detected in follicular fluid inside the female reproductive system, suggesting a relationship between oxidative imbalance, granulosa cell metabolism, and oocyte competency. Isofurans, which are more prevalent in the presence of elevated oxygen levels, may indicate oxidative stress in mitochondria and complications with cellular respiration. The current comprehension of lipid peroxidation indicators in infertility and assisted reproduction remains insufficient. This review aims to synthesise current information on isoprostanes and isofurans as reliable indicators of oxidative lipid damage in reproductive biology, highlighting their effects on gamete quality, mitochondrial dysfunction, and results in assisted reproduction. Our research seeks to clarify the biological importance of current experimental and clinical findings, highlighting their potential as clinically relevant biomarkers in reproductive medicine.

## 1. Introduction

Infertility affects a considerable proportion of couples of reproductive age worldwide and is recognised as a complex disorder influenced by genetic factors, epigenetic changes, metabolic health, environmental influences, lifestyle decisions, and other variables [1]. There is growing interest in the impact of oxidative stress on reproductive failure, since alterations in cellular redox balance influence several processes critical for fertilisation and embryonic development. Reactive oxygen species participate in physiological signalling inside the reproductive tract, influencing processes such as sperm capacitation, acrosome response, follicular development, and ovulation [2]. Disrupting the fragile equilibrium between oxidant-producing systems and protective mechanisms may lead to excessive oxidative activity, resulting in damage to nucleic acids, proteins, and membrane lipids [3]. Reproductive cells are particularly susceptible to this kind of damage due to their membranes being highly enriched with polyunsaturated fatty acids, hence increasing their vulnerability to lipid oxidation induced by free radicals [4].

Lipid peroxidation is a significant consequence of oxidative stress on reproductive organs. The process starts with the extraction of hydrogen atoms from polyunsaturated fatty acids in membrane phospholipids [5]. This initiates a cascade that generates reactive lipid radicals and subsequent oxidation products. These processes alter the membrane architecture, reduce fluidity, and disrupt the signalling pathways essential for cellular health. In spermatozoa, characterised by elevated concentrations of polyunsaturated fatty acids in their plasma membranes, oxidative lipid degradation may diminish motility, compromise membrane integrity, and increase susceptibility to DNA fragmentation [6]. Excessive lipid peroxidation in the ovarian milieu may adversely influence granulosa cell metabolism, mitochondrial activity in the oocyte, and intercellular communication within the follicular niche, eventually compromising oocyte competence and early embryonic development [7].

F2-isoprostanes are recognised as highly specific indicators of lipid peroxidation among the byproducts generated during free radical-mediated lipid oxidation. Prostaglandin-like compounds are generated by the non-enzymatic oxidation of arachidonic acid, which is esterified in membrane phospholipids, and then released into bodily fluids by phospholipases [8]. F2-isoprostanes, formed independently of cyclooxygenase activity, directly indicate oxidative events in vivo induced by free radicals. Their chemical stability and uniformity across many biological matrices have positioned them as one of the most reliable indicators of lipid peroxidation [9]. Investigations in reproductive medicine have shown elevated levels of F2-isoprostanes in the seminal plasma of infertile males, which correlate with diminished sperm motility and alterations in the sperm membrane structure. Detection in follicular fluid has been recorded, suggesting that oxidative lipid damage may influence the metabolic environment around the developing oocyte [10,11,12].

Isofurans represent an additional category of oxidation products derived from arachidonic acid. They exhibit a high degree of similarity. Isofurans are more readily produced in the presence of elevated oxygen levels, indicating a dysfunction in mitochondrial respiration [13]. Mitochondria serve as a primary generator of reactive oxygen species inside cells. Alterations in electron movement within mitochondria may facilitate the formation of isofurans over isoprostanes. These characteristics render isofurans particularly effective indicators of oxidative stress in mitochondria [14]. Mitochondrial dysfunction is becoming recognised as a crucial factor affecting reproductive ageing, reduced oocyte competence, and abnormal embryonic development [15,16]. The diminished effectiveness of oxidative phosphorylation, decreased adenosine triphosphate production, and the buildup of mitochondrial DNA damage might obstruct energy-dependent processes critical for meiotic spindle formation, chromosomal segregation, and initial embryonic development. Evaluating oxidative lipid products associated with mitochondrial dysfunction might provide a new understanding of the molecular pathways linking oxidative stress to reproductive failure [15].

Despite the increasing recognition of oxidative stress as a significant contributor to reproductive issues, research on isoprostanes and isofurans concerning infertility and assisted reproduction remains limited. Numerous studies examining oxidative damage in reproductive medicine include indirect indicators such as malondialdehyde, total antioxidant capacity, or comprehensive reactive oxygen species assessments [17]. These approaches may lack specificity and reproducibility, since they often exhibit varying oxidative processes and are assessed using assays susceptible to cross-reactivity and methodological discrepancies. An increased comprehension of these indicators may elucidate the oxidative mechanisms influencing gamete quality, fertilisation capacity, and embryo survival. Our analysis aims to examine the impact of oxidative lipid peroxidation, via isoprostanes and isofurans, on reproductive failure. This text specifically addresses three critical enquiries: (i) the manner in which these metabolites reflect and mediate oxidative stress within the reproductive microenvironment, (ii) their impact on mitochondrial function and cellular homeostasis in gametes, and (iii) their potential clinical significance as biomarkers for infertility and assisted reproduction.

Increasing evidence from human reproductive research indicates the significance of F2-isoprostanes. Τhe function of isofurans remains ambiguous in this context. Their inclusion in this study is based on their established biochemical relationship with oxygen tension and mitochondrial oxidative stress, both of which are very relevant to reproductive physiology.

## 2. Materials and Methods

A narrative review of the literature was conducted to synthesise current information about the involvement of isoprostanes and isofurans in infertility and assisted reproductive technologies. We performed a structured literature search of the biomedical literature using the PubMed/MEDLINE database. The literature search covered studies published from January 2000 to December 2025. The search strategy incorporated terminology related to oxidative lipid peroxidation and reproductive biology, including “isoprostanes,” “F2-isoprostanes,” “isofurans,” “oxidative stress,” “lipid peroxidation,” “male infertility,” “female infertility,” “follicular fluid,” “seminal plasma,” and “assisted reproduction.” To ensure adequate coverage of fundamental biochemical investigations and clinically pertinent reproductive research, we also screened the reference lists of selected articles to identify additional relevant studies. No formal date restrictions were applied during the initial screening. But the final selection reflects the defined timeframe.

Studies were selected for their topical relevance to oxidative lipid peroxidation and reproductive biology. Both experimental and clinical research were assessed, concentrating on those that investigate the generation, quantification, and biological relevance of isoprostanes and isofurans in reproductive situations. Studies not directly related to oxidative lipid damage in reproductive organs or lacking mechanistic or clinical relevance were removed from further analysis.

We excluded non-English literature, publications unrelated to oxidative lipid damage, and research not relevant to reproductive biology. The emphasis was on studies elucidating molecular mechanisms, biochemical routes, and clinical data that might improve understanding of the roles of isoprostanes and isofurans in reproductive functions and infertility. We then categorised the chosen literature by subject to construct a coherent narrative overview of oxidative lipid damage in the reproductive systems of both men and women and its implications for assisted reproduction.

## 3. The Biochemical Synthesis of Isoprostanes and Isofurans

### 3.1. Lipid Peroxidation and Oxidative Stress in Cellular Membranes

Isoprostanes are regarded as dependable indicators of lipid peroxidation, resulting from the non-enzymatic oxidation of polyunsaturated fatty acids. Their uniqueness must be contextualised within the overarching idea of oxidative stress, since they mostly indicate membrane lipid damage rather than encompassing all oxidative processes. Furthermore, analytical problems persist, including diversity in measurement methodologies, the potential for artefact creation during sample processing, and inconsistencies across biological matrices, which may influence comparability among investigations [18,19].

Lipid peroxidation typically starts when a highly reactive species, such as the hydroxyl radical, abstracts a hydrogen atom from a bis-allylic methylene group of a polyunsaturated fatty acid. The carbon-centred lipid radical rapidly interacts with molecular oxygen to form lipid peroxyl radicals [20]. The lipid bilayer sustains significant oxidative damage over time due to recurrent cycles of propagation. This process induces structural consequences that alter membrane fluidity, enhance permeability, and compromise the functional integrity of membrane-associated proteins. Lipid peroxidation generates several reactive secondary products that may alter proteins and nucleic acids, exacerbating cellular damage [21].

Reproductive cells have significant susceptibility to lipid peroxidation. The plasma membrane of spermatozoa is abundant in polyunsaturated fatty acids, particularly docosahexaenoic acid and arachidonic acid. These acids enhance membrane flexibility and facilitate the dynamic remodelling that transpires during capacitation and the acrosome response [22]. Oxidative damage to these lipids alters membrane fluidity and impairs ion-channel functionality, resulting in reduced sperm motility and diminished fertilisation capability. Lipid peroxidation in the ovarian follicle may affect granulosa cell membranes and mitochondrial architecture, thereby impairing steroidogenesis, cellular signalling, and metabolic connections between somatic cells and the developing oocyte [6]. Oxidative alterations in the follicular milieu may influence the advancement of meiosis, the assembly of spindles, and the maturation of the oocyte’s cytoplasm, therefore impacting the embryo’s developmental capacity [23].

### 3.2. Synthesis of F2-Isoprostanes from Arachidonic Acid

F2-isoprostanes are a distinct class of prostaglandin-like compounds generated by the oxidation of arachidonic acid by free radicals. Arachidonic acid is a polyunsaturated fatty acid of twenty carbon atoms. It is located at the sn-2 position of phospholipids in cellular membranes and serves as a precursor for several signalling molecules [8]. Arachidonic acid is enzymatically metabolised under normal physiological circumstances by cyclooxygenase, lipoxygenase, or cytochrome P450 pathways, leading to the production of prostaglandins, leukotrienes, and related lipid mediators. Oxidative stress initiates a non-enzymatic process that produces isoprostanes directly inside membrane phospholipids [24].

The synthesis of F2-isoprostanes begins when reactive radicals extract hydrogen from arachidonic acid. This generates a carbon-centred lipid radical that interacts with molecular oxygen to produce peroxyl intermediates. The intermediates undergo intramolecular cyclisation, yielding endoperoxide structures analogous to prostaglandin intermediates [25]. A complex assembly of stereoisomeric F2-isoprostanes is generated during reduction and rearrangement. These isoprostanes remain esterified in membrane phospholipids until they are degraded by phospholipases. These compounds originate from the oxidation of membrane-bound arachidonic acid, rather than via enzymatic processes involving free fatty acids. This indicates that their concentrations directly reflect oxidative damage occurring in cellular membranes [26].

F2-isoprostanes possess significant chemical stability. Isoprostanes, in contrast to several other lipid oxidation products that degrade rapidly or interact with other compounds, persist in biological fluids and tissues for an extended duration. This enables precise measurement with analytical techniques such as mass spectrometry [27]. Their stability and specificity have established them as among the most dependable indicators of oxidative stress in vivo. In reproductive biology, elevated amounts of F2-isoprostanes have been seen in the seminal plasma of infertile males, associated with reduced sperm motility and alterations in the sperm membrane structure [28]. Detection in follicular fluid suggests that lipid peroxidation in the ovarian milieu may influence oocyte competence and early embryonic development. Besides their diagnostic value, several isoprostane species have biological activity and may interact with prostanoid receptors, possibly influencing vascular tone, inflammatory signalling, and cellular stress responses in reproductive organs [29].

### 3.3. Isofurans and the Impact of Oxygen Tension and Mitochondrial Dysfunction

Isofurans represent a distinct category of oxidation products derived from arachidonic acid by lipid peroxidation induced by free radicals. Their synthesis begins via methods similar to those that generate isoprostanes. However, structural alterations throughout the oxidative cascade result in molecules with substituted tetrahydrofuran rings rather than the cyclopentane structures characteristic of isoprostanes [30]. The substantial reliance of isofuran synthesis on oxygen tension is a distinctive characteristic. Experimental investigations indicate that elevated oxygen levels facilitate the formation of isofurans, while diminished oxygen levels promote the formation of isoprostanes [13].

The alteration in response pathways responsive to oxygen significantly impacts cellular physiology, since elevated oxygen tension often indicates issues with mitochondrial respiration. Mitochondria are primarily responsible for the majority of cellular energy production. This occurs via oxidative phosphorylation, which relies on the transfer of electrons throughout the respiratory chain [31]. Electrons leaking from complexes I and III may partly reduce oxygen, generating superoxide radicals that may then convert into additional reactive oxygen species. When mitochondria malfunction, ineffective electron transport generates an excess of oxidants and increases the availability of oxygen in the vicinity. This facilitates the formation of isofurans during lipid peroxidation events [32].

The relationship between isofurans and oxidative stress in mitochondria is particularly significant in reproductive research. Oocytes possess a significant concentration of mitochondria that provide the energy required for meiotic spindle assembly, chromosomal segregation, and the first embryonic division. Mitochondrial malfunction associated with ageing, metabolic diseases, or environmental stress may reduce ATP generation while simultaneously increasing oxidative damage to mitochondrial lipids and DNA. The aggregation of this damage has been linked to reduced oocyte competence, abnormal chromosomal segregation, and developmental stagnation of embryos. The midpiece of spermatozoa contains mitochondria that provide energy for flagellar movement and create reactive oxygen species, which may initiate lipid peroxidation in adjacent membrane structures [16].

The identification of isofurans provides insights into oxidative mechanisms associated with mitochondrial dysfunction. When examined with isoprostanes, these compounds provide enhanced insights into the metabolic milieu of reproductive organs. Isoprostanes indicate generalised lipid peroxidation in cellular membranes, whereas isofurans may reflect oxidative conditions associated with impaired mitochondrial respiration. These indicators together improve the comprehension of oxidative lipid damage in gametes and reproductive organs, clarifying molecular pathways that may influence fertility and reproductive outcomes [25].

From a clinical standpoint, this distinction may be advantageous in elucidating the underlying oxidative milieu. Increased levels of isoprostanes may suggest lipid peroxidation at the outset, while an increased quantity of isofurans may suggest elevated oxygen tension and potential mitochondrial dysfunction. This may be essential when examining oxidative stress in reproductive tissues and in environments that utilise assisted reproduction.

## 4. Identification and Quantification of Isoprostanes and Isofurans

### 4.1. Analytical Methods for Quantifying Lipid Peroxidation Products

Accurately measuring lipid peroxidation products is challenging due to the structural similarities across various oxidation-derived compounds, which are often present in minimal quantities. The acknowledgement of isoprostanes as reliable markers of oxidative lipid damage led to the development of analytical techniques capable of distinguishing these molecules from enzymatically generated prostaglandins and other oxidised lipids derived from arachidonic acid metabolism [33]. Historically, immunological assays were often used to detect F2-isoprostanes in bodily fluids. Although these tests facilitated the first assessment of oxidative biomarkers in clinical research, subsequent investigations revealed considerable limitations. Structural similarities among various lipid oxidation products may cause cross-reactivity in antibody-based assays, resulting in an overestimation of concentrations or reduced analytical specificity [34].

Advancements in analytical chemistry have firmly established chromatographic methods combined with mass spectrometry as the preferred method for measuring isoprostanes and related oxidation products. Gas chromatography-mass spectrometry has long been the most effective method for detecting F2-isoprostanes [8]. The analytical technique often involves the extraction of lipids from biological samples, followed by the hydrolysis of esterified isoprostanes from phospholipids. Following purification procedures designed to isolate significant chemicals, derivatisation processes are often used to enhance volatility and increase chromatographic resolution [9]. Gas chromatography subsequently isolates the many stereoisomers produced during lipid peroxidation. Mass spectrometry subsequently detects each constituent according to its own fragmentation pattern. This technique for isolating and identifying structures enables precise quantification of certain isoprostane isomers derived from the oxidative alteration of arachidonic acid [8].

Liquid chromatography coupled with tandem mass spectrometry has emerged as a valuable analytical technique with several advantages in clinical and experimental contexts. Liquid chromatography techniques enable the direct analysis of biological materials after very simple extraction procedures. This differs from gas chromatography techniques, which may need many derivatisation processes [31]. Tandem mass spectrometry has high sensitivity and may detect chemicals present at minimal concentrations (picomolar). The use of multiple reaction monitoring enhances test specificity by identifying distinct fragmentation transitions associated with each lipid oxidation product. These novel methods enable the simultaneous measurement of many oxidised lipids within a single study. This has enabled a more comprehensive characterisation of oxidative lipid networks that extend beyond individual indicators [35].

From a biological perspective, precisely detecting these compounds is crucial due to the lipid oxidation cascade, which generates several structurally similar intermediates. Free radicals oxidising arachidonic acid may produce several regioisomeric and stereoisomeric products. This is contingent upon the location of oxygen addition and the mechanism of intramolecular cyclisation. Analytical techniques must, therefore, differentiate between substances with comparable molecular weights but distinct structural configurations [24]. Mass spectrometry techniques do this by using high-resolution fragmentation patterns that distinguish between various isoprostane families. Establishing a connection between oxidative lipid products and specific cellular activities, particularly in reproductive organs, requires a high degree of precision [36].

### 4.2. Biological Matrices Relevant to Reproductive Physiology

The selection of biological matrices for oxidative biomarker study profoundly influences the interpretation of lipid peroxidation assessments. Clinical investigations often use systemic compartments such as plasma and urine to assess overall oxidative stress [37]. Isoprostanes in the bloodstream indicate oxidative processes occurring in various organs, while isoprostanes in the urine provide a comprehensive assessment of lipid peroxidation products that have entered the bloodstream and subsequently been excreted by the kidneys. Urinary measures are often considered very accurate since they represent cumulative oxidative processes, minimising the influence of localised metabolic fluctuations or transient physiological changes [38].

Reproductive science has progressively shifted its attention to biological fluids that directly reflect the milieu around gametes. Seminal plasma is a complex amalgamation of fluids produced by the epididymis, prostate, seminal vesicles, and accessory glands. In this environment, spermatozoa confront both protective antioxidant systems and potential oxidative stresses [39]. The sperm plasma membrane has a high concentration of polyunsaturated fatty acids, enhancing its flexibility but increasing its susceptibility to lipid degradation. Reactive oxygen species generated by leukocytes, immature spermatozoa, or mitochondrial function inside the sperm midpiece might provoke oxidative processes that affect these lipids. Assessing F2-isoprostanes in seminal plasma provides insights into oxidative alterations that impact sperm membrane integrity and cellular activity [40].

The ovarian follicle represents an additional biological compartment in which oxidative activities may influence reproductive outcomes. Follicular fluid surrounds the developing oocyte and indicates metabolic connections among granulosa cells, theca cells, and the oocyte [41]. This milieu comprises a combination of metabolites, steroid hormones, growth factors, and compounds that regulate redox processes, together orchestrating oocyte development. The mitochondrial metabolism of granulosa cells significantly influences the local oxidative environment, since steroidogenesis and cellular respiration generate reactive oxygen species as byproducts [42]. Excessive oxidative activity relative to antioxidant capacity in a specific region might lead to lipid peroxidation events in cellular membranes or lipoprotein particles within follicular fluid. The presence of isoprostanes in this region indicates oxidative circumstances that may impact the quality and developmental potential of oocytes [43].

Investigations on oxidative damage have also examined more reproductive organs. Researchers have examined placental tissues, amniotic fluid, and endometrial samples to investigate the potential impact of oxidative stress on complications during pregnancy. Although these biological matrices originate from advanced phases of reproductive physiology, their examination aids in comprehending oxidative lipid damage throughout the reproductive continuum. The presence of isoprostanes or isofurans in these tissues often indicates alterations in mitochondrial metabolism, inflammatory signalling, or vascular function in reproductive organs.

### 4.3. Biochemical Advantages Compared to Conventional Oxidative Stress Biomarkers

In reproductive biology, oxidative stress has previously been assessed with several indicators. Malondialdehyde and 4-hydroxynonenal are two prevalent biomarkers. Both are byproducts of lipid peroxidation. Aldehydes are generated through fragmentation reactions following extensive oxidative damage to polyunsaturated fatty acids. Their detection may signify lipid oxidation; nevertheless, the metabolic mechanisms that generate these compounds are complex and may include many lipid substrates [44]. Furthermore, the analytical techniques used to detect these aldehydes, particularly thiobarbituric acid-reactive substance tests, sometimes lack specificity and may identify other compounds with similar chemical characteristics.

Conversely, isoprostanes and isofurans originate directly from the free radical oxidation of arachidonic acid, making them more precise indicators of lipid peroxidation in membrane phospholipids. They develop independently of the enzymes responsible for prostaglandin synthesis, hence facilitating the distinction between oxidative damage and inflammatory signalling mechanisms [45]. Another significant characteristic is their structural stability. Numerous reactive lipid aldehydes rapidly interact with proteins or nucleic acids; nevertheless, isoprostanes maintain chemical stability in biological fluids, allowing for precise measurement even post-sample collection and storage [46].

The molecular insights provided by these biomarkers provide an additional advantage. Quantifying isoprostanes indicates the extent of lipid peroxidation occurring in cell membranes, while the synthesis of isofurans is influenced by oxygen levels and the redox status of the mitochondria. When examined together, these compounds provide enhanced insights into the oxidative milieu of cells [13]. This knowledge is particularly significant in reproductive biology, where mitochondrial function is crucial for sperm motility and oocyte development. Oxidative damage to mitochondrial membranes or electron transport complexes may hinder energy generation, alter redox-sensitive signalling pathways, and compromise the cellular processes critical for fertilisation and embryonic development [47].

The quantification of isoprostanes and isofurans, enabled by the integration of sensitive analytical methods with physiologically relevant biomarkers, has become an essential tool for investigating oxidative processes in reproductive tissues. Identifying them provides a molecular insight into lipid oxidation processes occurring in gametes and the reproductive microenvironment. This may elucidate the molecular processes linking oxidative stress to infertility and reproductive failure.

## 5. Isoprostanes in Male Infertility

Given the growing evidence linking lipid peroxidation to sperm dysfunction, key human studies evaluating seminal F2-isoprostanes in male infertility are summarised in Table 1.

### 5.1. The Composition of Lipids in the Sperm Membrane and Their Susceptibility to Oxidative Stress-Induced Damage

Spermatozoa have one of the most specialised plasma membranes among mammalian cells. This membrane has highly dynamic lipid domains that facilitate activities such as capacitation, hyperactivation, the acrosome reaction, and ultimately fusion with the oocyte plasma membrane [51]. Polyunsaturated fatty acids, particularly docosahexaenoic acid and arachidonic acid, are prevalent in sperm membranes. These fatty acids possess many double bonds that provide the requisite flexibility to bend and reorganise the membrane during fertilisation. This flexibility facilitates the rapid alteration of lipid microdomains and the relocation of membrane proteins implicated in signal transduction [52].

The biochemical characteristics of lipid peroxidation processes increase the susceptibility of sperm membranes to oxidative damage. Removing hydrogen from polyunsaturated fatty acids results in lipid radicals that rapidly react with molecular oxygen to form lipid peroxyl intermediates. These intermediates initiate chain reactions by targeting adjacent lipids inside the membrane bilayer. This results in progressively enlarging regions of oxidative damage. As the process progresses, the membrane’s integrity diminishes. Oxidative alterations to membrane phospholipids modify the organisation of the bilayer, disrupt lipid-protein interactions, and affect the distribution of cholesterol inside the membrane. Such alterations hinder the membrane’s ability to maintain the precise shape required for sperm capacitation and the regulated influx of calcium ions that initiates hyperactivated motility [53].

Proteins contained in the lipid bilayer malfunction when the membrane lipids are compromised. Ion channels, transporters, and receptors need the surrounding lipids to maintain their appropriate conformation and function well. Oxidative alterations to adjacent phospholipids may modify the membrane’s viscosity and disrupt the spatial configuration of these proteins. This may impede sperm from activating the requisite signalling pathways [6]. The generation of secondary lipid oxidation products produces reactive electrophiles capable of forming covalent adducts with membrane proteins. Such alterations may impair the molecular apparatus governing flagellar motion and render the axonemal complex less stable [54].

Another reason spermatozoa exhibit diminished strength is their limited antioxidant presence. During spermiogenesis, the majority of the cytoplasm is eliminated, resulting in a diminutive cell with few enzymatic defence mechanisms. In comparison to somatic cells, the body has a reduced quantity of enzymes, including superoxide dismutase, catalase, and glutathione peroxidase [6]. Spermatozoa heavily rely on the antioxidant components of seminal plasma for protection against oxidative damage. Excessive reactive oxygen species, such as those produced during leukocyte activation, the retention of cytoplasmic residues in immature spermatozoa, or mitochondrial activity in the sperm midpiece, disrupt the equilibrium between oxidants and antioxidants. Under these circumstances, lipid peroxidation may rapidly occur, compromising the structural integrity and functionality of the sperm membrane [55].

The identification of F2-isoprostanes as products of free radical-mediated oxidation of arachidonic acid has provided researchers with a robust method to quantify oxidative damage in biological membranes. These compounds originate from lipid peroxidation processes occurring in membrane phospholipids [8]. Their presence in seminal plasma indicates that oxidative processes are impacting sperm membranes and other lipid-rich structures within the reproductive tract. Isoprostanes are a more definitive indicator of oxidative damage induced by free radicals compared to many other secondary lipid oxidation products arising from other metabolic processes [28].

Clinical investigations examining oxidative biomarkers in semen have consistently shown elevated amounts of F2-isoprostanes in men with abnormal semen parameters. Individuals with asthenozoospermia, oligoasthenoteratozoospermia, and other forms of idiopathic male infertility have elevated amounts of these chemicals [28]. The correlation between isoprostane levels and sperm motility is particularly intriguing. The axonemal architecture and the signalling pathways associated with the membrane must collaborate to regulate calcium flux and phosphorylation processes in the flagellum. Oxidative alterations to membrane lipids may disrupt these processes, hence impeding flagellar motion and diminishing progressive motility efficacy [6].

Certain isoprostane species function as biomarkers and possess biological activity that may alter cellular signalling. Specific constituents of this molecular family interact with prostanoid receptors on vascular and epithelial cells, altering the body’s inflammatory response and vascular tone. The precise function of these signalling systems in the male reproductive tract remains ambiguous. It is conceivable that isoprostanes produced in this region may influence blood flow in the testes or epididymis. Alterations in blood circulation or inflammatory signalling in the region may also modify the oxidative milieu around developing spermatozoa [56].

Measuring isoprostanes in seminal plasma not only indicates the presence of oxidative stress but also elucidates the molecular consequences of lipid oxidation inside the reproductive system. Elevated levels may indicate that oxidative damage is pervasive, impacting membrane architecture, protein function, and cellular signalling pathways. This information may be important when a guy has difficulties conceiving, while normal semen analysis reveals no apparent issues. Assessing oxidative biomarkers may reveal biochemical issues impacting sperm function, despite normal morphological features.

### 5.2. The Impact on Sperm DNA Integrity and Mitochondrial Function

Oxidative stress on spermatozoa influences not only membrane lipids but also DNA inside the nucleus and mitochondria. During spermiogenesis, chromatin undergoes extensive compaction as histones are substituted with protamines. This renders the nuclear structure very compact, safeguarding the paternal genome while it traverses the male reproductive canal [6]. Although this configuration safeguards the DNA in sperm, it remains susceptible to damage from oxidative stress when reactive oxygen species accumulate inside the cell. Oxidative alterations to nucleotides may induce base modifications, abasic sites, and strand breakage, hence diminishing genomic stability. This kind of damage is associated with a reduced likelihood of conception, delayed embryo development, and an increased risk of miscarriage [57].

Lipid peroxidation products may inflict molecular damage on DNA via several mechanisms. Upon the oxidation of polyunsaturated fatty acids, reactive aldehydes may migrate from the membrane milieu into the nucleus and interact with nucleic acids. These electrophilic compounds engage with DNA bases, inducing alterations that inhibit normal replication and transcription. Oxidative stress may activate signalling pathways that induce apoptosis in sperm cells, resulting in DNA fragmentation and reduced sperm viability [58].

Mitochondrial activity significantly influences sperm quality and is very susceptible to oxidative circumstances. The mitochondrial sheath inside the sperm provides energy to the flagella by oxidative phosphorylation, enabling their movement [59]. Electrons traversing the respiratory chain sometimes escape from complexes I and III, resulting in the conversion of molecular oxygen into superoxide radicals. Antioxidant enzymes typically prevent the accumulation of reactive species. Nevertheless, excessive oxidative stress may compromise these protective mechanisms. Lipid peroxidation in mitochondrial membranes alters the configuration of respiratory complexes and the mitochondrial membrane potential, hence impeding sperm motility by hindering ATP production [4].

The accumulation of oxidative damage in mitochondria may initiate a cascade of metabolic issues that impair sperm function. Reduced ATP levels impede the prolonged motility of flagella, whereas alterations in mitochondrial membrane potential may initiate apoptotic signalling pathways. The measurement of isoprostanes in seminal plasma is often associated with indicators of mitochondrial dysfunction, including reduced mitochondrial membrane potential and impaired sperm motility. Oxidative lipid damage and mitochondrial dysfunction are interrelated processes that may contribute to male infertility [60].

These chemical processes illustrate how oxidative stress may adversely affect several aspects of sperm physiology when they interact synergistically. Lipid peroxidation alters membrane structure and signalling pathways, reactive lipid derivatives damage nuclear DNA, and mitochondrial malfunction restricts the energy supply required for mobility. Isoprostanes in seminal plasma serve as a biochemical marker for oxidative processes, providing insight into the molecular environment around spermatozoa. Examining these indicators may enhance our understanding of the oxidative mechanisms that impair male fertility and assist in identifying individuals who might benefit from targeted antioxidant or metabolic therapies.

## 6. Isoprostanes and Female Infertility 

Evidence regarding the role of isoprostanes in the female reproductive system, particularly within the follicular microenvironment, remains limited but clinically relevant, as summarised in Table 2.

Compared to the male reproductive domain, evidence about isoprostanes in female reproduction is rather scarce and inconsistent. The current study demonstrates that oxidative stress may be assessed both systemically and locally within the follicular environment. Preliminary results demonstrated the presence of isoprostanes in follicular fluid, supporting the hypothesis that lipid peroxidation may occur within the ovarian milieu. Some investigations have shown associations between elevated follicular or systemic isoprostane levels and reduced ovarian reserve, impaired oocyte yield, and lower fertility. Findings on embryo quality remain equivocal, highlighting the complex interplay between oxidative stress, compensatory antioxidant pathways, and follicular dynamics. Overall, our findings endorse a physiologically plausible, albeit not completely elucidated, function of isoprostanes in female fertility and IVF results.

### 6.1. Oxidative Stress in the Ovarian Microenvironment

Folliculogenesis occurs in a metabolically active ovarian milieu characterised by continuous biochemical communication between somatic cells and the developing oocyte. The granulosa cells, theca cells, and oocyte function together as a unified metabolic entity to regulate steroidogenesis, nutrient exchange, and intracellular signalling essential for oocyte maturation [63]. Granulosa cells generate reactive oxygen species as byproducts of mitochondrial respiration, steroid hormone production, and enzymatic oxidation reactions during their regular metabolic processes. Superoxide dismutase, glutathione peroxidase, catalase, and non-enzymatic antioxidants constitute the body’s intrinsic defences that maintain redox equilibrium within the follicular compartment [64]. The regulated generation of reactive oxygen species is crucial to signalling pathways that govern follicular development, meiotic resumption, and ovulation. This indicates that oxidant molecules may function as both signalling mediators and potential agents of cellular harm [65]. Evidence regarding the role of isoprostanes in the female reproductive system, particularly within the follicular microenvironment, remains limited but clinically relevant, as summarised in Table 2.

Alterations in redox homeostasis modify the chemical composition of the follicular milieu and initiate oxidative processes that damage proteins, lipids, and nucleic acids. The polyunsaturated fatty acids present in the membrane phospholipids of granulosa cells and oocytes are very susceptible to oxidative alteration [65]. The extraction of hydrogen from the bis-allylic methylene groups in these fatty acids initiates lipid peroxidation events, propagating via the formation of lipid radicals and peroxyl intermediates. Lipid oxidation products accumulate over time, altering membrane organisation, modifying membrane-associated proteins, and affecting the structure of intracellular organelles. Oxidative damage to mitochondrial membranes in granulosa cells may inhibit steroidogenic pathways that convert cholesterol into oestrogenic hormones via cytochrome P450-dependent enzymatic processes [66].

Numerous metabolic interactions occur inside the ovarian follicle, facilitated by gap junctions linking granulosa cells to the oocyte. These junctions facilitate the transfer of metabolites such as pyruvate, amino acids, and nucleotides across cells. These metabolites facilitate the growth and maturation of oocytes [67]. Oxidative injury to granulosa cell membranes or mitochondrial metabolism may disrupt these metabolic exchanges. The oocyte may be incapable of sustaining energy production and maintaining redox equilibrium inside the cell if it fails to transfer metabolic substrates effectively. Consequently, oxidative alterations in the follicular niche may influence both nuclear and cytoplasmic maturation processes that impact developmental competence [23].

The function of mitochondria significantly influences oocyte physiology. Mature oocytes contain many mitochondria distributed throughout the cytoplasm. The mitochondria provide the energy required for the assembly of the meiotic spindle and the segregation of chromosomes [68]. Oxidative damage to mitochondrial membranes may impede the electron transport chain and reduce the synthesis of adenosine triphosphate. Insufficient energy hinders the normal assembly of the meiotic spindle machinery, increasing the likelihood of chromosomal misalignment during meiosis [16]. Lipid peroxidation events occurring in mitochondrial membranes further destabilise the inner membrane structure that facilitates oxidative phosphorylation. Such alterations diminish mitochondrial efficiency, a phenomenon seen in ageing women and those with ovarian dysfunction.

### 6.2. Identification of Isoprostanes in Follicular Fluid

Follicular fluid serves as a biochemical reservoir that reflects the metabolic and endocrine activities of the follicular unit. When plasma-derived chemicals infiltrate the follicle, they amalgamate with secretory products generated by granulosa and theca cells [69]. This generates an intricate amalgamation of hormones, lipids, metabolites, cytokines, and growth factors. Analysing follicular fluid provides valuable insights into the molecular milieu around the developing oocyte. Lipid peroxidation products derived from cellular membranes accumulate in this compartment and may be detected using very sensitive analytical techniques. F2-isoprostanes have emerged as highly specific indicators of oxidative lipid degradation inside the ovarian follicle [70].

Reactive oxygen species initiate the non-enzymatic oxidation of arachidonic acid residues embedded in membrane phospholipids, resulting in the formation of F2-isoprostanes. Intramolecular cyclisation events generate prostaglandin-like endoperoxide intermediates that then undergo conformational changes to yield a series of stereoisomeric F2-isoprostanes [8]. These molecules remain esterified in membrane phospholipids until phospholipase enzymes degrade them, releasing them into the extracellular environment. Their presence in follicular fluid indicates that lipid peroxidation occurs in the membranes of granulosa cells, inside mitochondrial structures, and in the lipoprotein particles located in the follicular compartment [71].

Elevated isoprostane levels in follicular fluid indicate that oxidative circumstances may alter follicular function. Lipid peroxidation in granulosa cell membranes may alter the arrangement of membrane receptors and transport proteins, which are crucial for steroid hormone synthesis and nutrient uptake [29]. Oxidative alterations to phospholipids may modify the physical characteristics of cell membranes, affecting their permeability and altering their morphology. Alterations to the structure may influence the vesicular trafficking mechanisms responsible for transporting cholesterol and other lipids essential for steroidogenesis [72].

Lipid peroxidation events occurring inside lipoproteins in follicular fluid may alter the biochemical composition of the follicular environment. Lipoprotein particles transport cholesterol and other lipids essential for hormone synthesis [73]. Upon oxidation, these particles may generate reactive lipid species capable of binding to membrane receptors or intracellular signalling pathways. The accumulation of these oxidation products may alter the inflammatory condition of the follicle and the communication between somatic cells and the oocyte [74].

The synchronised maturation of nuclear and cytoplasmic elements dictates the developmental capability of an egg. Successful fertilisation and optimal early embryonic development need meticulous regulation of mitochondrial metabolism, cytoskeletal architecture, and intracellular signalling networks [75]. Oxidative damage to cellular lipids may inhibit these activities via several molecular mechanisms. Lipid peroxidation in mitochondrial membranes alters the assembly of electron transport complexes and destabilises the electrochemical gradient essential for oxidative phosphorylation. When the respiratory chain functions suboptimally, it produces less ATP and permits more electron leakage. This increases the production of reactive oxygen species and perpetuates oxidative stress [76].

Alterations in mitochondrial metabolism influence many critical events occurring during oocyte maturation. The construction of the meiotic spindle requires energy-dependent polymerisation of microtubules and meticulous coordination of centrosomal components. Insufficient ATP may compromise spindle stability and result in improper chromosome alignment [16]. A primary cause of aneuploidy in human oocytes is chromosomal missegregation resulting from improper spindle formation. Oxidative modifications to proteins that facilitate spindle organisation further destabilise the meiotic apparatus.

Lipid oxidation products may influence intracellular signalling pathways that regulate calcium levels and cytoskeletal remodelling. Calcium waves occurring during fertilisation initiate oocyte activation and trigger a series of actions essential for embryonic development [77]. Oxidative alterations to membrane channels or their associated regulatory proteins may disrupt these signalling pathways. Reactive lipid intermediates generated during peroxidation processes may bind to proteins that modulate the actin cytoskeleton. This may alter the framework that facilitates cell division [78].

Accumulation of oxidative damage in the follicular environment may influence embryo development prior to fertilisation. The oocyte’s developmental capacity is diminished due to alterations in mitochondrial metabolism, altered signalling pathways, and oxidative modifications to cellular components. Assessing isoprostanes in follicular fluid provides a biochemical perspective on these molecular processes by indicating the extent of lipid peroxidation within the follicular niche.

## 7. Mitochondrial Dysfunction and Isofurans in Reproductive Biology

It is crucial to acknowledge that, in contrast to isoprostanes, there exists less direct human evidence about isofurans in reproductive tissues or in contexts involving assisted reproduction. Thus, the following discourse is mostly based on recognised biochemical and mitochondrial concepts, with careful application to reproductive biology.

### 7.1. Oxygen-Dependent Lipid Peroxidation Pathways

Isofurans are produced using the same oxidative mechanisms as isoprostanes; however, their synthesis is significantly influenced by the oxygen levels in the cellular microenvironment. When free radicals oxidise arachidonic acid, the resultant peroxyl radicals throughout the lipid chain may exhibit varying mobility contingent upon the local oxygen concentration. In conditions of reduced oxygen, cyclisation processes yielding cyclopentane structures, prevalent in F2-isoprostanes, occur with greater frequency [79]. Elevated oxygen levels induce rearrangements that produce substituted tetrahydrofuran ring structures known as isofurans. We can distinguish various oxidative situations in organisms by examining the oxidation of lipids under different oxygen levels [80].

The biochemical milieu in which these processes transpire is essential for ascertaining the fate of lipid oxidation products. Due to an abundance of oxygen and the cells’ inability to use electrons effectively, isofuran is likely to be generated inside cellular compartments [81]. Inadequate reduction of molecular oxygen generates reactive oxygen species that interact with membrane phospholipids rich in polyunsaturated fatty acids. This initiates the formation of lipid radicals. The extent of lipid peroxidation is influenced by the surrounding oxygen concentration, which influences the stability and configuration of lipid peroxyl intermediates [20]. An increased concentration of oxygen enhances the stability of peroxyl radicals, facilitating processes that yield oxidation products, including tetrahydrofuran. Thus, an increased presence of isofuran indicates a more oxidative environment, characterised by elevated oxygen levels and the continuous generation of radicals [82].

Metabolic dysfunctions that impede mitochondrial function may provide biochemical environments conducive to isofuran formation in reproductive organs. Mitochondrial oxidative phosphorylation sustains ovarian follicles, spermatozoa, and early embryos by generating energy and maintaining intracellular redox equilibrium [83]. Mitochondria use diminished oxygen when their functionality is impaired, resulting in an increase in intracellular oxygen levels. In such settings, the biochemical milieu facilitates the formation of isofurans during the degradation of lipids in mitochondrial membranes and next to phospholipid structures. Examining isofurans may enhance our understanding of oxidative mechanisms specifically associated with mitochondrial dysfunction, rather than oxidative stress in a broader context [84].

### 7.2. Mitochondrial Origins of Reactive Oxygen Species

When metabolism is dysregulated, mitochondria significantly contribute to the production of reactive oxygen species inside cells. In oxidative phosphorylation, electrons derived from metabolic substrates traverse a sequence of protein complexes embedded in the inner mitochondrial membrane. These complexes facilitate the transfer of electrons to molecular oxygen via a sequence of processes [85]. They also provide a proton gradient that facilitates ATP production. When functioning optimally, the respiratory chain efficiently facilitates electron transport. However, some electrons may fail to reach the terminal oxidase complex from complexes I and III. This leads to the partial reduction of molecular oxygen and the generation of superoxide radicals.

Superoxide dismutase rapidly converts superoxide, generated in the mitochondrial matrix, into hydrogen peroxide. Subsequently, hydrogen peroxide may traverse mitochondrial membranes or integrate into intracellular redox signalling pathways. When antioxidant defences are insufficient, hydrogen peroxide may convert into highly dangerous hydroxyl radicals. Fenton-type reactions involving transition metals result in this occurrence. These radicals efficiently attack polyunsaturated fatty acids in mitochondrial membranes, triggering lipid peroxidation events that spread across the lipid bilayer [86].

Mitochondrial membranes are particularly susceptible to oxidative damage due to their high content of cardiolipin, a unique phospholipid that maintains the integrity of respiratory chain complexes. The oxidation of cardiolipin modifies the formation of these complexes and obstructs electron transport. This results in an increased leakage of electrons, hence generating a greater quantity of reactive oxygen species. Lipid peroxidation processes begin in these circumstances, producing various oxidation products, such as isoprostanes and isofurans [87].

The relationship between mitochondrial oxidative stress and reproductive biology is notably important. Oocytes require operational mitochondria to get the energy essential for meiotic maturation and first embryonic cleavage [16]. Spermatozoa need mitochondria in the midpiece to generate ATP, essential for flagellar motility. Consequently, dysfunctions in mitochondrial operations significantly impact an individual’s reproductive capacity. The identification of lipid peroxidation products associated with mitochondrial membranes serves as a significant biochemical indicator of such issues.

### 7.3. Isofurans as Biomarkers of Mitochondrial Oxidative Stress in Gametes

The molecular relationship between oxygen levels and isofuran synthesis has rendered these molecules more significant as markers of oxidative stress in mitochondria. When mitochondria malfunction, they emit increased levels of oxygen into the surrounding environment. This increases the likelihood that lipid peroxidation processes will yield isofurans [9]. Measuring these chemicals may indicate oxidative processes primarily associated with mitochondrial failure rather than broad radical generation inside the cell.

Mitochondrial metabolism inside the ovarian follicle underpins many pathways that promote oocyte development. Granulosa cells use oxidative phosphorylation to generate the metabolic precursors required for steroid hormone synthesis and energy production [63]. Malfunctioning mitochondria in these cells may alter the chemical composition of the follicular fluid, increasing the susceptibility of membrane lipids to oxidative damage. The presence of isofuran in the follicular compartment may signify problems with mitochondrial respiration affecting both the developing oocyte and the granulosa cells. Isofurans may clarify metabolic abnormalities that might affect reproductive potential, given the importance of mitochondrial function in oocyte survival [88].

Spermatozoa have metabolic traits that make mitochondrial oxidative stress especially critical. Oxidative phosphorylation is the mechanism that provides the mitochondrial sheath of the midpiece with energy for flagellar motility. Excessive reactive oxygen species in this compartment may induce lipid peroxidation in mitochondrial membranes and adjacent plasma membrane structures [6]. It alters the mitochondrial membrane potential and reduces ATP generation when oxidative damage accumulates in these membranes. ATP is essential for sustained locomotion. The generation of isofuran during these oxidative processes may signify mitochondrial malfunction, compromising sperm functioning [6].

Mitochondrial oxidative stress may affect early embryonic development after fertilisation, transcending the gametes. During the early stages of development, embryos depend on mitochondrial metabolism supplied from the egg to facilitate cellular division [89]. Oxidative damage to mitochondrial DNA or membrane lipids may hinder cellular energy generation and affect the signalling pathways critical for embryonic development. Measuring lipid peroxidation products associated with mitochondrial membranes may enhance our understanding of the oxidative environment in which embryos develop [15].

Oxidation of lipids results in the production of isofurans, which serve as biochemical indicators of oxidative conditions marked by increased oxygen levels and mitochondrial malfunction. In reproductive biology, where mitochondrial function is essential for gamete viability and embryonic development, the discovery of these molecules might clarify metabolic abnormalities that may hinder reproductive success [90]. Simultaneously analysing isoprostanes and isofurans offers a more thorough insight into the oxidative processes inside reproductive organs. This may assist us in understanding how mitochondrial dysfunction might lead to infertility.

## 8. Clinical Implications in Assisted Reproduction

### 8.1. Oxidative Lipid Damage as a Contributor to Gamete Competence

Reactive oxygen species and their associated lipid peroxidation products have a situationally dependent effect in reproductive biology. Physiological levels are crucial for processes including sperm capacitation, ovulation, and intracellular signalling. High or uncontrolled oxidative activity is linked to impaired gamete function and negative reproductive consequences. This dual function illustrates the need to preserve redox equilibrium, rather than only seeing oxidative stress as harmful. Assisted reproductive technologies rely on the successful interaction between viable gametes and a favourable biochemical environment that promotes early embryonic development. Oxidative stress is getting more and more attention as a factor that affects the quality of gametes, especially when normal clinical tests cannot explain why reproduction does not go well [91]. Lipid peroxidation in gamete membranes is a crucial molecular phenomenon linking oxidative imbalance to diminished reproductive capacity. Oxidative modification of polyunsaturated fatty acids in the membranes of spermatozoa and oocytes can significantly impact membrane structure, signalling pathways, and intracellular metabolic processes [6].

In the context of male infertility, oxidative damage to sperm membranes impairs the molecular mechanisms that regulate motility and fertilisation. Capacitation involves substantial reorganisation of the sperm plasma membrane, marked by alterations in cholesterol distribution, increased membrane fluidity, and the activation of signalling pathways that control calcium influx [92]. Lipid peroxidation disrupts these processes by modifying membrane phospholipids and destabilising the microdomains that organise ion channels and receptors. Alterations in the membrane structure impede the necessary transformations for fertilisation to occur. The measurement of isoprostanes in seminal plasma serves as a molecular indicator of oxidative damage that undermines sperm membrane integrity and functional capacity [28].

Oxidative lipid damage may influence oocyte maturation and embryonic development within the ovaries. Moving mitochondria, putting together spindles, and separating chromosomes are all very important steps in the last stages of oocyte maturation [93]. Lipid peroxidation in cellular membranes can alter mitochondrial structure and disrupt intracellular signalling pathways essential for these processes. Elevated concentrations of lipid oxidation products in follicular fluid may signify metabolic conditions that impede oocyte developmental competence. Finding these kinds of biomarkers could tell us a lot about the biochemical environment around the oocyte when it is taken out for assisted reproduction [29].

### 8.2. The Potential of Biomarkers in Assessing the Reproductive Microenvironment

A significant objective in reproductive medicine is to identify dependable biomarkers of oxidative damage. Conventional metrics of oxidative stress, including evaluations of reactive oxygen species or total antioxidant capacity, provide only indirect insights into cellular oxidative mechanisms [94]. Conversely, isoprostanes and isofurans are directly produced from the free radical-mediated oxidation of membrane phospholipids, thereby offering a more accurate depiction of lipid peroxidation processes occurring in vivo. Quantifying these molecules in reproductive biological fluids may provide a more precise assessment of the oxidative conditions impacting gametes [9]. However, without validated thresholds and clinically tested predictive models, their current use remains primarily investigational. The practical use of these biomarkers is hindered by the absence of defined diagnostic criteria and established performance indicators, including sensitivity, specificity, and area under the curve values. Subsequent research should focus on delineating these factors for their integration into standard therapeutic practice.

Examining seminal plasma for lipid peroxidation products has been proposed as a supplementary diagnostic approach in the evaluation of male infertility. Elevated isoprostane concentrations are associated with diminished sperm motility and increased DNA fragmentation [49]. This implies that assessing these biomarkers may reveal oxidative alterations that standard semen analysis fails to detect. This kind of information could help find patients who could benefit from targeted treatments that aim to restore redox balance in the reproductive tract.

In instances of female infertility, the analysis of follicular fluid offers a direct understanding of the metabolic environment surrounding the developing oocyte. Finding lipid peroxidation products in this compartment could mean that oxidative conditions are present that could affect the maturation of oocytes and the potential for embryos to develop. Elevated isoprostane levels in follicular fluid may indicate dysfunction in mitochondrial metabolism, inflammatory signalling, or steroidogenic pathways within the follicular unit. Including oxidative biomarkers in the examination of follicular physiology may enhance our comprehension of factors influencing reproductive outcomes in assisted reproduction cycles.

### 8.3. Effects on Treatment Methods Focusing on Oxidative Stress

The recognition of oxidative stress as a contributor to reproductive dysfunction has sparked interest in therapeutic strategies aimed at restoring redox equilibrium. Scientists have looked into giving antioxidants as a possible way to lower oxidative damage to gametes in a number of clinical settings. Scientists have studied vitamins C and E, coenzyme Q10, N-acetylcysteine, and other polyphenolic molecules to see if they can stop reactive oxygen species and improve the function of mitochondria. However, clinical outcomes remain inconsistent, and evidence from larger studies and meta-analyses has not consistently demonstrated improvements in live birth rates [95].

Despite a compelling scientific rationale, the efficacy of antioxidant supplementation for infertility remains ambiguous. Multiple systematic reviews and meta-analyses have shown diverse or little impact on essential reproductive outcomes, particularly live birth rates. This disparity may stem from variations in patient selection, baseline oxidative state, kind and dosage of antioxidants administered, and the methodological design of the trials. Therefore, antioxidant-based therapies should be treated cautiously, since their consistent use in assisted reproduction lacks substantial empirical evidence.

Understanding the molecular mechanisms of lipid peroxidation may aid in the development of more targeted interventions. Mitochondrial dysfunction is a major source of reactive oxygen species in reproductive cells, so strategies that improve mitochondrial function may be very helpful. Substances that enhance mitochondrial electron transport, strengthen antioxidant defence mechanisms, or stabilise mitochondrial membranes may reduce the generation of lipid peroxidation byproducts in gametes. Measuring isoprostanes and isofurans may serve as diagnostic biomarkers and indicators for evaluating the effectiveness of therapeutic interventions designed to mitigate oxidative damage [96].

Adding oxidative biomarkers to assisted reproductive medicine could make infertility treatment more personalised in the long run. By identifying patients for whom oxidative lipid damage is a significant factor, clinicians can tailor therapeutic strategies that address the underlying molecular abnormalities affecting reproductive function. Continuing research on the clinical significance of isoprostanes and isofurans may provide substantial insights into the role of oxidative stress in assisted reproduction and promote the development of innovative strategies to improve reproductive outcomes [97].

In order to assess the treatment response and redox equilibrium, clinical investigations in assisted reproduction have employed specific oxidative stress biomarkers, such as isoprostanes and associated lipid peroxidation products, to evaluate antioxidant interventions [10,12].

## 9. Discussion

The current literature reveals a consistent biological pattern: oxidative damage to membrane lipids is not a secondary effect of reproductive dysfunction but a crucial event linking redox imbalance to impaired gamete quality, reduced fertilisation potential, and unsatisfactory outcomes in assisted reproduction. In the examined research, F2-isoprostanes are the most analytically robust lipid peroxidation products identified in reproductive fluids. Conversely, extensive oxidative panels often have more variable clinical associations. Compelling evidence arises from seminal plasma, where oxidative lipid damage regularly corresponds with sperm motility, morphology, viability, immaturity, and inflammatory reproductive disease. Research on follicular fluid is limited and diverse. However, it corroborates the notion that oxidative lipid remodelling occurs with ovarian ageing, follicular dysfunction associated with PCOS, and metabolic issues connected to endometriosis. The prevailing hypothesis is that F2-isoprostanes do not act as a general predictor of reproductive success; instead, they operate as a physiologically relevant signal of membrane-centric oxidative stress within specific reproductive microenvironments. To provide a concise overview of the available human evidence, the principal clinical studies evaluating F2-isoprostanes in male and female reproductive settings are summarised in Table 3.

Lin et al. were the pioneers in demonstrating that 8,12-iso-iPF2α may be quantified in follicular fluid during in vitro fertilisation [10]. Lipid peroxidation occurs directly inside the ovarian microenvironment rather than only in the general oxidative state of the organism. Rosen et al. substantiated this concept by showing that isoprostanes in follicular fluid rise with a decrease in egg production and are more closely linked to ovarian ageing, as shown by ovarian response, rather than only chronological age [11]. Kim et al. extended the discussion beyond IVF cohorts by showing an inverse relationship between circulating F2-isoprostanes and AMH in a population-based sample, especially among younger reproductive ages [61]. This suggests that oxidative lipid stress may influence ovarian reserve before the clinical onset of substantial reproductive loss. Schisterman et al. complicated the biology by demonstrating that endogenous reproductive hormones influence F2-isoprostane levels [98]. Oestradiol had a beneficial impact, contrary to the often anticipated antioxidant profile in reproductive endocrinology. Nobles et al. presented a periconceptional viewpoint, revealing that heightened preconception urine isoprostanes are linked to decreased fecundability, but raised levels during early gestation correspond with a lower chance of pregnancy loss [62]. This discovery questions any naive view of isoprostanes as universally harmful across all reproductive phases.

This observed temporal variation may indicate that oxidative stress has distinct effects at various stages of reproductive biology. Elevated isoprostane levels before conception may indicate a dysfunctional oxidative environment, potentially impairing the oocyte’s fertilisation capacity or the early embryo’s viability. Conversely, the elevation seen in early pregnancy may indicate a regulated, normal oxidative response associated with implantation, trophoblast invasion, and the first phases of placental development. The findings indicate that F2-isoprostanes should not be regarded as static markers of oxidative damage. Ιnstead, they should be seen as dynamic indicators whose biological and clinical relevance may fluctuate based on reproductive stage and metabolic setting.

Fujimoto et al. investigated follicular oxidative biology through a thorough analytical framework, evaluating lipid peroxidation derivatives and antioxidant enzyme activities in individual follicles. Nonetheless, they did not establish a clear correlation with embryo morphology parameters, such as cell number and fragmentation score [12]. Gongadashetti et al. similarly discovered that women with PCOS had elevated levels of follicular fluid ROS, total antioxidant capacity (TAC), and 8-isoprostane compared to women with tubal-factor controls [99]. Several methodological and clinical reasons may explain these discrepancies. Differences in patient demographics, including age, ovarian reserve status, and previous diseases such as PCOS, might significantly influence oxidative profiles in the follicular environment. Furthermore, variations in the analytical techniques used to assess oxidative biomarkers, particularly the preference for immunoassays over mass spectrometry-based approaches, may explain the discrepancies across research. The time of sample collection is crucial, since signs of oxidative stress may fluctuate during the ovarian stimulation cycle. Furthermore, diversity in regulated ovarian stimulation procedures, including various gonadotropin regimens and GnRH analogues, may act as confounding factors affecting oxidative balance and reproductive results.

Nonetheless, they could not identify significant correlations between these levels and the quantity of oocytes recovered, the cleavage rate, or the grading of embryos. In a comprehensive analysis of follicular fluid composition in PCOS, Moreira et al. discovered oxidative stress as part of a larger pathogenic profile that includes inflammatory mediators, hormonal abnormalities, and altered growth-factor signalling [100]. Zec et al. investigated redox homeostasis in follicular fluid from a bench-to-bedside perspective, contending that the follicular oxidative state cannot be reduced to a single marker, as the oocyte develops within a precisely regulated microenvironment shaped by both pro-oxidant and antioxidant influences [101]. Brinca et al. elucidated this notion in endometriosis by showing that oxidative stress, immunological activation, and altered energy metabolism coexist in follicular fluid, serum, and plasma, associated with reduced oocyte and embryo quality [69].

The male reproductive literature exhibits more internal consistency and defines a more defined pathophysiological function for F2-isoprostanes. Khosrowbeygi et al. documented markedly increased concentrations of seminal free 8-isoprostane in instances of asthenozoospermia, asthenoteratozoospermia, and oligoasthenoteratozoospermia, coupled with inverse connections to sperm motility and morphology [102]. Zarghami et al. similarly discovered that sperm motility had a negative correlation with both 15-F2α-isoprostane and malondialdehyde, despite the isoprostane disparity between asthenozoospermic and normozoospermic males not achieving statistical significance [102]. This indicates that functional decline may correlate more reliably with oxidative stress than with categorical diagnostic classifications. Collodel et al. subsequently established that infertile men with varicocele and idiopathic infertility present elevated levels of seminal F2-isoprostanes, alongside reduced concentration, impaired motility, suboptimal morphology, decreased viability, and increased rates of apoptosis, necrosis, and immaturity [17]. Signorini et al. synthesised these data by proposing that isoprostanes in male infertility signify not only oxidative stress but also alterations in sperm fatty acid content, reduced antioxidant defence, and compromised sperm maturation [56]. Moretti et al. further confirmed their results in a larger cohort, revealing that seminal F2-isoprostanes negatively correlate with morphology, viability, total progressive motility, and quick motility, while also identifying a clinically significant threshold of 29.96 ng/mL [49].

Collodel et al. further elucidated that oxidative lipid degradation is localised rather than uniformly distributed, occurring in particular regions of spermatozoa [50]. Collodel et al. demonstrated the immunolocalization of 8-iso-prostaglandin F2α in the midpiece and cytoplasmic remnants, anatomical areas critically linked to mitochondrial function and compromised sperm maturation [50]. Moretti et al. later confirmed altered localisation patterns in idiopathic infertile males, demonstrating enhanced signals extending to the acrosome, midpiece, and tail in those with a higher F2-isoprostane load and less progressive motility [49]. Chen et al. enhanced the biochemical research by demonstrating that diverse PUFA-derived metabolites, including several eicosanoid-related substances, distinguish fertile from infertile males, even among normozoospermic people [77]. Barati et al. enhanced the extensive oxidative framework by asserting that male infertility cannot be explained just by membrane damage, since oxidative stress simultaneously affects DNA oxidation, gene expression alteration, and epigenetic regulation during spermatogenesis [103].

The relationship between oxidative lipid damage and sperm chromatin requires careful examination, since it offers a more complex understanding than a simple “higher is worse” framework. Collodel et al. found that seminal F2-isoprostanes positively correlated with double-stranded DNA sperm and negatively correlated with mature sperm chromatin [17]. Moreover, couples with good ART results and high-quality embryos had increased levels of F2-isoprostanes in comparison to control groups. Moretti et al. demonstrated that in cases of idiopathic infertility, men surpassing the oxidative threshold displayed reduced progressive motility and increased levels of resolvin D1, a lipid mediator involved in inflammation resolution, suggesting a possible co-elevation of oxidative and counter-regulatory pathways [49]. Moretti et al. conducted an inflammatory-based analysis revealing that F2-isoprostanes were associated with both MDA and IL-1β, indicating a more accurate distinguishing of idiopathic infertility relative to MDA [49]. These data indicate that F2-isoprostanes are not only passive markers of permanent damage. At intermediate altitudes, they may signify an active metabolic condition marked by the simultaneous presence of membrane remodelling, inflammatory signalling, and compensatory resolution mechanisms. The more credible interpretation is biphasic: moderate increases may occur alongside physiological or semi-adaptive lipid turnover, while prolonged elevations, especially in inflammatory conditions like varicocele and genitourinary infection, indicate membrane destabilisation and clinically significant oxidative damage.

The inflammatory aspect is elucidated by the biology of phospholipase. Collodel et al. established a favourable link between cytosolic phospholipase A2 and seminal F2-isoprostanes, especially in instances of varicocele and leukocytospermia [17]. Moretti et al. later showed that F2-isoprostanes, MDA, and IL-1β rise together in inflammatory male infertility circumstances and collectively distinguish fertile from infertile males [49]. Halliwell and Lee clarify the methodological and biochemical framework for interpreting these findings, emphasising that F2-isoprostanes are among the most dependable indicators of lipid peroxidation when evaluated through mass spectrometry, while also warning that biological matrix, sampling strategy, and interpretation can substantially affect conclusions [104]. The Halliwell and Lee framework is particularly advantageous in comparison to the frameworks of Collodel et al. and Moretti et al. [17,28,104]. To detect oxidative damage in semen with sufficient specificity to differentiate between idiopathic infertility and inflammatory reproductive disease, F2-isoprostanes are superior to generic tests such as TBARS-derived MDA, provided the analytical technique is robust. The preeminence of F2-isoprostanes compared to MDA in 2025 is corroborated by the Moretti research, which is consistent with reproductive data and the comprehensive literature on oxidative stress [28].

The difference between male and female data is particularly illuminating from a molecular standpoint. Lin et al., Rosen et al., and Gongadashetti et al. concur that oxidative lipid stress exists in follicular fluid [10,11,100]. The clinical implications are less definitive than those seen in semen. Fujimoto et al. found no association between most follicular oxidative indicators and embryo shape [12]. Conversely, Brinca et al. and Moreira et al. situate oxidative imbalance within a broader pathogenic setting concerning endometriosis and PCOS [69,100]. Khosrowbeygi et al., Collodel et al., and Moretti et al. repeatedly demonstrate strong correlations between seminal F2-isoprostanes and sperm functionality [17,49,102]. A structural reason is that sperm membranes have a high concentration of polyunsaturated fatty acids and possess a limited amount of antioxidants in their cytoplasm, rendering them more susceptible to damage from peroxidation. A further reason is functional: sperm motility is directly contingent upon the integrity of membrane fluidity, ion-channel activity, and mitochondrial ATP synthesis; hence, oxidative membrane damage rapidly results in observable phenotypic alterations. Follicular fluid, on the other hand, is a heterogeneous compartment that includes contributions from granulosa cells, plasma transudate, immunological mediators, metabolic substrates, and the developing oocyte. Oxidative signals in such an environment are indeed there, but they are more dispersed and less likely to indicate a singular binary outcome.

In contrast to the generally consistent findings in male reproductive research, the data in female reproductive biology are more varied and lack clear mechanistic understanding. This likely pertains to the complexity of the follicular milieu, where several interacting variables, including metabolic, hormonal, and inflammatory pathways, alter oxidative equilibrium. Therefore, oxidative indicators in female circumstances may not immediately correspond with consistent functional results, requiring a more cautious, context-sensitive interpretation.

One last consideration is what remains absent. The majority of the information provided here pertains to F2-isoprostanes, while isofurans are mostly absent, despite their potential significance in mitochondrial oxidative stress under elevated oxygen conditions. Signorini et al. and Barati et al. implicitly suggest that mitochondrial redox imbalance is a key feature of male infertility, whereas Brinca et al., Moreira et al., and Zec et al. emphasise energetic and oxidative anomalies within the ovarian environment [56,69,100,101,103]. The subsequent logical progression involves not just increasing the frequency of biomarker measurements, but also enhancing the understanding of lipid oxidation mechanisms inside reproductive organs. Chen et al. are progressing in lipidomics [77]. Nonetheless, there is a lack of genuine integration of oxidised PUFA species, classical isoprostanes, inflammatory mediators, and clinical reproductive outcomes.

The material analysed here supports a complex interpretation rather than a unique linear conclusion. Lin et al., Rosen et al., Fujimoto et al., Gongadashetti et al., Brinca et al., Moreira et al., Zec et al., Kim et al., Schisterman et al., and Nobles et al. collectively illustrate that oxidative lipid remodelling in female reproduction indicates a compromised follicular and reproductive milieu, although direct forecasts of ART success remain inconsistent [11,12,61,62,69,98,99,100,101,105]. Khosrowbeygi et al., Collodel et al., Moretti et al., Signorini et al., and Chen et al. provide compelling evidence that seminal F2-isoprostanes signify clinically relevant oxidative damage affecting sperm motility, membrane integrity, maturation, chromatin organisation, and, in certain contexts, ART outcomes [17,28,56,77,102]. The most plausible synthesis asserts that F2-isoprostanes are not simply vague indicators of “oxidative stress” but rather specific markers of membrane-focused oxidative biology, with their reproductive significance dependent on tissue context, concentration gradient, inflammatory state, and inherent metabolic condition. Conversely, a particular database does not yet support the development of reliable clinical performance indicators, including prediction models or verified cut-off values. Rather than indicating a lack of therapeutic promise, this restriction is a reflection of the variability and preliminary nature of the available data. To connect mechanistic knowledge with therapeutic application, prospective trials including standardised designs and well-specified diagnostic and prognostic goals are necessary.

**Figure 1:**
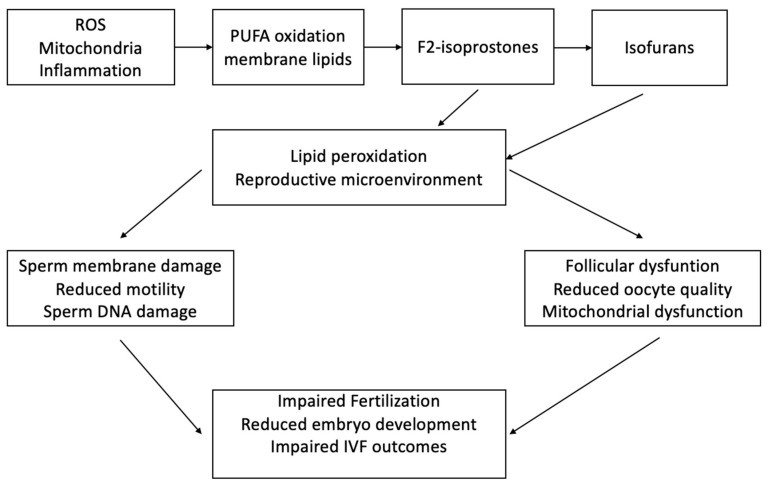
Oxidative stress-induced lipid peroxidation and its impact on human reproductive function. ROS, originating from mitochondrial dysfunction and inflammation, induce the oxidation of polyunsaturated fatty acids in cell membranes, resulting in the formation of F2-isoprostanes and isofurans. Lipid peroxidation products indicate oxidative damage in reproductive microenvironments. In the male reproductive system, they are linked to sperm membrane impairment, decreased motility, and DNA fragmentation. An oxidative imbalance in the follicular milieu of the female reproductive system leads to granulosa cell impairment, reduced oocyte viability, and mitochondrial malfunction. All of these alterations are associated with reduced probabilities of conception, complications in embryonic development, and adverse outcomes from ART. Created by the authors.

The mechanistic and clinical interplay between oxidative stress, lipid peroxidation, F2-isoprostanes, isofurans, and reproductive dysfunction is summarised in Figure 1.

Although biologically significant, the therapeutic utility of isoprostanes and isofurans is limited due to the absence of standardised testing techniques or established criteria for patient classification or clinical decision-making.

## 10. Limitations of the Current Evidence

Even though there is more and more research on isoprostanes in reproductive biology, the evidence we have is still scattered and not always consistent. The majority of studies examining the correlation between lipid peroxidation products and infertility have depended on relatively limited patient cohorts, frequently confined to particular clinical populations, including varicocele, idiopathic male infertility, or designated subgroups of women undergoing assisted reproduction. Consequently, the generalisability of numerous observations is still restricted. There is still a lack of larger multicentre studies with standardised inclusion criteria, especially when it comes to female infertility and assisted reproductive technologies.

Another significant limitation stems from the substantial variability in the analytical methodologies employed to quantify isoprostanes. Different studies have used enzyme immunoassays, high-performance liquid chromatography, or mass spectrometry-based methods, each of which has its own level of sensitivity and specificity. Mass spectrometry is still the best way to find F2-isoprostanes, but a lot of clinical studies still use immunoassays, which may give higher concentrations than they really are because they can react with other compounds that are similar in structure. This methodological diversity hinders direct comparisons between datasets and constrains the formulation of universally recognised reference values.

Understanding how oxidative biomarkers relate to reproductive outcomes is also very hard. Numerous studies have investigated the associations between isoprostane concentrations and semen parameters, embryo morphology, or pregnancy outcomes. But infertility is caused by a number of things, including hormones, metabolism, genetics, and the environment. In this intricate biological framework, oxidative stress markers seldom function as autonomous indicators of reproductive success. For instance, studies on oxidative stress in follicular fluid have yielded inconclusive results concerning their relationships with embryo development and implantation potential. Certain datasets indicate correlations with miscarriage or reduced ovarian reserve, while others do not identify significant associations with embryo morphology or fertilisation rates.

The temporal dimensions of oxidative stress measurement constitute another unresolved concern. The majority of existing studies evaluate biomarkers at a singular time point, generally during oocyte retrieval or semen collection. Oxidative processes in reproductive tissues are very dynamic and can change when hormones are present, when inflammation signals are present, when metabolism is high, or when the environment changes. Consequently, individual measurements yield merely a constrained representation of a multifaceted biochemical milieu. Longitudinal studies investigating oxidative lipid mediators across various treatment cycles or during folliculogenesis would yield a more precise depiction of their biological significance.

Finally, our current understanding of isofurans in reproductive biology is still very limited. Isofurans are established indicators of lipid peroxidation that occur preferentially in high oxygen tension environments; however, their presence and physiological relevance in reproductive fluids have not been thoroughly examined. Due to the pivotal role of mitochondrial metabolism in gamete function, the lack of studies investigating isofurans in follicular fluid, seminal plasma, or embryo culture media signifies a significant deficiency in the field.

Another limitation is the potential impact of pre-analytical variables on the quantification of lipid peroxidation biomarkers. The levels of isoprostanes and isofurans that are measured may be altered by sample handling, storage temperature, freeze–thaw cycles, and processing delays. This is particularly critical for biological matrices such as seminal plasma and follicular fluid, which are frequently collected in a variety of clinical settings and lack standardised processing methods.

A further constraint is the difficulty in quantifying isofuran in clinical reproductive specimens. Isofurans are seldom assessed compared to F2-isoprostanes and need very sensitive mass spectrometry methods, which may not be uniformly available in various studies. Moreover, matrix-specific constituents in biological fluids, such as follicular fluid or seminal plasma, may introduce variability and impede accurate measurement. The absence of defined reference ranges for isofurans in reproductive tissues limits their clinical use and complicates the interpretation of present results. Future research should explicitly examine isofuran production in reproductive tissues and fluids to see whether their mechanistic significance correlates with clinically relevant indicators.

A significant constraint of the existing research is the absence of reliable clinical performance data, including verified cut-off values and prediction models. In the absence of these measurements, the transition from biochemical indicators to clinically applicable tools is insufficiently realised.

## 11. Conclusions

Research on oxidative lipid metabolites has increasingly clarified the importance of lipid peroxidation in reproductive physiology and infertility. Increasing research indicates that F2-isoprostanes are among the most well-established biomarkers of lipid peroxidation and oxidative damage in reproductive organs. Research on seminal plasma indicates that elevated levels of F2-isoprostane are consistently associated with impaired sperm function, including reduced motility, morphological alterations, increased immature sperm, and inflammatory reproductive disorders. Research findings suggest that oxidative lipid mediators may reflect changes in membrane fatty acid composition and mitochondrial metabolism, both essential for sperm maturation and fertilisation potential.

Investigations into follicular fluid indicate that lipid peroxidation occurs within the ovarian milieu during assisted reproduction. The detection of isoprostanes in follicular fluid highlights the presence of oxidative processes inside the developing follicle, a biochemical environment that directly influences oocyte maturation and embryonic developmental potential. Although the relationship between embryo shape and pregnancy outcomes remains equivocal, existing studies indicate that oxidative lipid signalling may influence follicular physiology in diseases such as polycystic ovary syndrome, diminished ovarian reserve, and endometriosis.

Isoprostanes are physiologically active lipid mediators that may alter inflammatory pathways, mitochondrial function, and cellular signalling networks. They transcend mere biomarkers. Their production results from the free radical oxidation of arachidonic acid inside membrane phospholipids. This links oxidative stress to alterations in membrane integrity, lipid signalling pathways, and gamete viability. These processes highlight a complex metabolic system in which lipid peroxidation intersects with inflammation, mitochondrial failure, and reproductive ageing.

Despite these advancements, many questions remain unanswered. Standardised analytical methods and larger clinical cohorts are essential to establish clinically relevant reference ranges for oxidative lipid metabolites in reproductive fluids. Furthermore, the potential involvement of supplementary lipid peroxidation products, particularly isofurans, requires further examination in reproductive organs. Given that isofurans mostly arise under conditions marked by elevated oxygen tension and mitochondrial oxidative stress, their investigation may enhance comprehension of the effects of mitochondrial dysfunction on infertility.

The investigation of oxidative lipid mediators represents a novel domain within reproductive biology research. The integration of lipidomic indicators into clinical reproductive medicine may improve the understanding of gamete physiology and promote the development of more precise diagnostic and treatment strategies for infertility.

## Figures and Tables

**Table 1:** Role of seminal F2-isoprostanes in male infertility and sperm dysfunction.

Study	Population	Sample	Biomarkers	Method	Main Findings	Clinical Implication
Khosrowbeygi and Zarghami, 2007, [48]	Men with abnormal semen parameters	Seminal plasma	Free 8-isoprostane	Immunoassay	Elevated isoprostane levels associated with reduced motility and abnormal morphology	Reflects oxidative damage to sperm membrane lipids
Collodel et al., 2021, [17]	49 infertile couples undergoing IVF/ICSI	Seminal plasma	F2-isoprostanes	GC/NICI-MS/MS	Positive correlation with double-stranded DNA and embryo quality; negative with chromatin maturity	Indicates role in sperm chromatin integrity and embryo development
Moretti et al., 2022, [49]	Infertile men with varicocele, infection, idiopathic infertility	Seminal plasma	F2-isoprostanes	GC/NICI-MS/MS	Defined pathological range and threshold values	Supports diagnostic use in clinical andrology
Moretti et al., 2025, [28]	46 infertile vs. 11 fertile men	Seminal plasma	F2-isoprostanes, MDA, IL-1β	GC-MS, HPLC, ELISA	Strong correlation between oxidative markers; F2-isoprostanes outperform MDA in distinguishing infertility subtypes	Superior biomarker of lipid peroxidation in inflammatory infertility
Collodel et al., 2015–2018, [50]	Men with infertility	Seminal plasma/sperm	F2-isoprostanes	GC-MS	Association with lipid peroxidation, sperm immaturity, and oxidative damage	Mechanistic link between ROS and sperm dysfunction

Studies on humans that look at seminal F2-isoprostanes in men who cannot have children. The table shows that they are linked to sperm motility, morphology, chromatin integrity, inflammatory conditions, and their possible use as biomarkers of lipid peroxidation in clinical andrology.

**Table 2:** F2-isoprostanes in female reproduction, follicular fluid, and IVF outcomes.

Study	Population	Sample	Biomarker	Method	Main Findings	Clinical Implication
Lin et al., 2005, [10]	29 women undergoing IVF	Follicular fluid	8,12-iso-iPF2α	LC-MS	Higher levels observed in non-pregnant patients; measurable in FF	Demonstrates feasibility of measuring oxidative stress directly in follicular microenvironment
Rosen et al., 2009, [11]	31 women undergoing ART	Follicular fluid	F2-isoprostanes	GC-MS	Higher levels associated with reduced oocyte number and ovarian ageing	Links oxidative stress with diminished ovarian reserve
Kim et al., 2020, [61]	830 late reproductive-age women	Plasma	F2-isoprostanes	Plasma biomarker analysis	Higher levels associated with lower AMH	Indicates systemic oxidative stress impacts ovarian reserve
Nobles et al., 2023, [62]	1228 women attempting conception	Urine	Multiple isoprostanes	Urinary biomarker analysis	Higher preconception levels associated with lower fecundability	Supports role of oxidative stress in early reproductive success
Fujimoto et al., 2011, [12]	39 IVF patients	Follicular fluid	Lipid peroxidation products	HPLC	No strong association with embryo quality detected	Suggests complexity of oxidative pathways in follicular environment

The table summarises the research on F2-isoprostanes and lipid peroxidation in female reproductive research, including their correlation with ovarian reserve, oocyte yield, and reproductive outcomes.

**Table 3:** Main human studies evaluating F2-isoprostanes in reproductive settings.

Study	Study Population	Sample	Biomarker(s)	Analytical Method	Main Findings	Clinical Implication
Khosrowbeygi and Zarghami, 2007/2008, [48]	Men with normozoospermia, oligozoospermia, asthenozoospermia, and teratozoospermia	Seminal plasma	Free 8-isoprostane and its hydrolysed form	Immunoassay-based measurement	Free 8-isoprostane showed inverse associations with sperm motility and morphology; higher lipid peroxidation was linked to abnormal semen quality	Supports the use of isoprostanes as markers of sperm membrane oxidative injury
Collodel et al., 2021, [17]	49 infertile couples undergoing IVF/ICSI	Seminal plasma	F2-isoprostanes	GC/NICI-MS/MS	Seminal F2-isoprostanes correlated positively with double-stranded sperm DNA and negatively with mature sperm chromatin; mildly increased levels were associated with better embryo quality in some cases	Indicates that seminal F2-isoprostanes may reflect both oxidative balance and sperm metabolic competence in ART settings
Moretti et al., 2022, [49]	147 infertile men with varicocele, urogenital infection, or idiopathic infertility, and 45 fertile controls	Seminal plasma	F2-isoprostanes	GC/NICI-MS/MS	Established a pathological range and proposed a cut-off distinguishing physiological from abnormal seminal F2-isoprostane levels	Strengthens the clinical applicability of seminal F2-isoprostanes as a diagnostic oxidative stress biomarker
Moretti et al., 2025, [28]	46 infertile men with varicocele, genitourinary infection, or idiopathic infertility, and 11 fertile controls	Seminal plasma	F2-isoprostanes, MDA, IL-1β	GC/NICI-MS for F2-isoprostanes; HPLC for MDA; ELISA for IL-1β	F2-isoprostanes, MDA, and IL-1β were all increased in inflammatory infertility states, but F2-isoprostanes discriminated pathological groups more accurately than MDA, especially idiopathic infertility	Supports F2-isoprostanes as a more informative marker of semen lipid peroxidation than MDA in inflammatory male infertility
Kim et al., 2020, [61]	830 late reproductive-age women from the CARDIA cohort	Plasma	F2-isoprostanes	Plasma biomarker analysis in epidemiologic cohort	Higher circulating F2-isoprostanes were associated with lower AMH levels, especially at younger reproductive ages	Suggests a link between systemic oxidative stress and diminished ovarian reserve
Nobles et al., 2023, [62]	1228 women attempting conception in the EAGeR trial	Urine	8-iso-PGF2α, 2,3-dinor-iPF2α-III, 5-iso-PGF2α-VI, 8,12-iso-iPF2α-VI	Urinary isoprostane measurement	Higher preconception isoprostane levels were associated with lower fecundability; early gestational levels rose thereafter and showed a complex association with pregnancy loss	Supports the relevance of isoprostanes as peripheral biomarkers of redox status in natural fertility and early pregnancy

Main human studies evaluating F2-isoprostanes in reproductive settings. The table includes primary human studies assessing isoprostanes in seminal plasma, blood, or urine in relation to semen quality, assisted reproduction outcomes, ovarian reserve, fecundability, and early pregnancy.

## Data Availability

No new data were created or analysed in this study. Data sharing is not applicable to this article.
